# Optimal Timing of Vericiguat Therapy in Patients with Heart Failure with Reduced/Mildly Reduced Ejection Fraction for Improving Mortality and Heart Failure Re-Hospitalization Rate

**DOI:** 10.3390/jcm14165856

**Published:** 2025-08-19

**Authors:** Yuki Hida, Teruhiko Imamura, Koichiro Kinugawa

**Affiliations:** Second Department of Internal Medicine, University of Toyama, Toyama 930-0194, Japan

**Keywords:** hemodynamics, guideline-directed medical therapy, hospitalization

## Abstract

**Background**: Vericiguat, a soluble guanylate cyclase stimulator, reduces cardiovascular events in patients with heart failure with reduced ejection fraction following clinical deterioration against guideline-directed medical therapy. However, the optimal timing for initiating vericiguat remains unclear. **Methods**: We retrospectively analyzed heart failure patients with reduced/mild-reduced ejection fraction who received vericiguat between 2021 and 2025 upon optimal guideline-directed medical therapy. The primary outcome was a composite of all-cause mortality and heart failure hospitalization. Patients were stratified by the number of prior heart failure hospitalizations (<2 vs. ≥2), and outcomes were assessed using multivariable Cox regression and biomarker trajectories over 6 months. **Results**: A total of 43 patients (with a median age of 73 years, 35 were men) were included. Of these, 26 (60%) patients had ≥2 prior hospitalizations. A number of hospitalizations ≥ 2 independently predicted the primary outcome (hazard ratio: 8.43; 95% confidence interval: 1.79–39.7; *p* = 0.007). Only patients with <2 prior hospitalizations showed significant improvements in plasma B-type natriuretic peptide levels (*p* = 0.049) and left ventricular ejection fraction (*p* = 0.016). In contrast, no meaningful biomarker changes were observed in patients with ≥2 hospitalizations. **Conclusions**: A history of two or more heart failure hospitalizations is a strong predictor of poor outcomes during vericiguat therapy. These findings suggest that initiating vericiguat earlier—before recurrent hospitalizations—may yield greater clinical benefit.

## 1. Introduction

Heart failure with reduced ejection fraction remains a significant global health challenge, imposing a substantial burden of morbidity and mortality despite major therapeutic advancements [1,2]. The current standard of care, known as guideline-directed medical therapy (GDMT), involves the foundational use of four drug classes: renin–angiotensin system inhibitors (especially angiotensin receptor neprilysin inhibitors), beta-blockers, mineralocorticoid receptor antagonists, and sodium–glucose co-transporter 2 inhibitors [1]. Despite this “quadruple therapy”, patients remain at significant residual risk of both worsening heart failure and mortality, particularly high-risk individuals [3,4]. Worsening heart failure is a critical condition characterized by clinical deterioration that necessitates urgent therapeutic escalation and is associated with a markedly high risk of re-hospitalization and mortality, underscoring the urgent need for additional therapeutic strategies [4,5].

Vericiguat, a novel oral soluble guanylate cyclase stimulator, has emerged as a promising agent for this very population. In heart failure, endothelial dysfunction and oxidative stress reduce the bioavailability of nitric oxide, leading to impaired function of the critical nitric oxide–soluble guanylate cyclase–cyclic guanosine monophosphate signaling pathway, which regulates vascular tone and myocardial function [6,7]. Vericiguat directly and indirectly stimulates soluble guanylate cyclase, independent of nitric oxide availability, thereby increasing cyclic guanosine monophosphate levels and promoting beneficial effects, including vasodilation and anti-fibrotic effects, and anti-inflammatory action [7,8]. The landmark VICTORIA (Vericiguat Global Study in Subjects with Heart Failure with Reduced Ejection Fraction) trial validated this approach, demonstrating that vericiguat reduced the composite endpoint of cardiovascular death or first heart failure hospitalization in heart failure with reduced ejection fraction patients who had recently experienced a worsening event despite receiving GDMT [9].

Despite its proven efficacy, the “optimal timing” for initiating vericiguat remains a critical and unresolved question. The VICTORIA trial enrolled a high-risk cohort with recent clinical deterioration, leading to a common perception that vericiguat should be reserved for patients who remain symptomatic or are re-hospitalized despite optimized foundational GDMT, a stance reflected in the current guidelines [1,10]. However, this reactive strategy of intervening only after significant disease progression may limit the drug’s potential benefits. Indeed, subgroup analyses from the VICTORIA trial suggested a greater benefit in patients with relatively lower baseline NT-pro BNP levels, implying that intervention in a less advanced disease state might be more effective [9].

We hypothesized that recurrent heart failure hospitalizations may serve as a surrogate marker for a more advanced, less treatment-responsive disease state in which the efficacy of vericiguat could be diminished. Therefore, this study aimed to investigate the impact of the number of prior heart failure hospitalizations on clinical outcomes in patients receiving vericiguat, to explore whether an earlier therapeutic intervention—before a patient establishes a history of recurrent admissions—confers a greater clinical benefit.

## 2. Methods

### 2.1. Study Design

This retrospective study included patients who received vericiguat therapy at a single large academic institute between September 2021 and February 2025.

Eligible patients were adults diagnosed with heart failure with reduced/mildly reduced LVEF (LVEF < 50%) who remained symptomatic despite receiving maximally tolerated doses of GDMT, including beta-blockers, renin–angiotensin system inhibitors (including angiotensin receptor neprilysin inhibitors), mineralocorticoid receptor antagonists, and sodium–glucose co-transporter 2 inhibitors. In addition, the included patients had experienced a recent episode of worsening heart failure, such as an urgent clinic visit for the up-titration of diuretics or heart failure medications, or hospitalization for intravenous diuretic therapy. Patients were required to have at least 6 months of available follow-up data after vericiguat initiation.

Patients were excluded if they were intolerant to vericiguat, had significant valvular heart disease requiring surgical or percutaneous intervention, or had a history of recent acute coronary syndrome or cardiac surgery within the past 3 months. We also excluded patients whose baseline or follow-up data were incomplete.

The independent variable was the number of prior heart failure hospitalizations. Day 0 was defined as the time when vericiguat therapy was initiated. The primary outcome was a composite of all-cause mortality and heart failure hospitalization during vericiguat therapy. The secondary outcome was the trajectory of key clinical parameters over a 6-month period. All patients provided written informed consent prior to enrollment. The study protocol was approved by the Institutional Ethics Committee.

### 2.2. Vericiguat Therapy

Patients generally received comprehensive quadruple therapy as tolerated according to the guidelines’ recommendation. The presence of residual symptoms and/or a recent episode of clinical worsening (e.g., hospitalization or intravenous diuretics) was the basis for considering vericiguat initiation. Vericiguat therapy was commenced only after patients had achieved clinical stabilization. Specifically, vericiguat was introduced either during routine outpatient follow-up or immediately following discharge from hospital, but only after patients had transitioned to oral diuretics, were clinically euvolemic, and demonstrated hemodynamic stability during background heart failure therapy. Vericiguat was never initiated during the acute phase of decompensation.

Treatment began at 2.5 mg/day, with the dose doubled at intervals of at least two weeks, under close monitoring of symptoms and vital signs. Therapy was discontinued in cases of symptomatic hypotension or other drug-related adverse events. Patients with prior use of soluble guanylate cyclase stimulators were not prescribed vericiguat. Follow-up was conducted at our institution or affiliated centers by board-certified cardiologists. Other heart failure medications were also adjusted according to the patients’ symptoms and examination findings at the discretion of the attending cardiologists. Heart failure-related examinations were scheduled in a standard manner during the vericiguat therapy, including chest X-rays, electrocardiograms, laboratory tests, and echocardiography.

### 2.3. Clinical Data Collection

Baseline demographics, comorbidities, medication history, laboratory results, and echocardiographic data were collected at the time of vericiguat initiation. Laboratory and echocardiographic parameters were also updated during the 6-month treatment period.

### 2.4. Statistical Analysis

All analyses were conducted using SPSS Statistics 26 (IBM, Armonk, NY, USA). Two-tailed *p*-values < 0.05 were considered statistically significant. Continuous variables were recorded as medians and interquartile ranges and compared using the Mann–Whitney U test. Categorical variables were expressed as counts (percentages) and compared using Fisher’s exact test.

The primary outcome was defined as a composite of all-cause death and heart failure hospitalization. The secondary outcome was the trajectory of plasma B-type natriuretic peptide (BNP), estimated glomerular filtration rate (eGFR), and left ventricular ejection fraction (LVEF) over six months.

The independent variable—the number of prior heart failure hospitalizations—was categorized and dichotomized based on a time-dependent receiver operating characteristic analysis. A cutoff value of ≥2 hospitalizations was statistically determined, yielding an area under the curve of 0.83.

Cox proportional hazard ratio regression analysis was used to assess the prognostic impact of this variable on the primary outcome. Multivariable adjustment was performed for variables with *p* < 0.010 in univariable analysis. Variables that were included in the univariable analyses were pre-defined according to their potential prognostic impacts. The cumulative incidence of the primary outcome was compared between the two groups, and it was divided by the cutoff of prior heart failure hospitalization instances using the log-rank test.

Changes in clinical parameters were assessed using the Wilcoxon signed-rank test, and between-group comparisons were evaluated with the Mann–Whitney U test.

## 3. Results

### 3.1. Baseline Characteristics

A total of 43 patients were included (Table 1). The median age was 73 (60, 79) years; 35 (81%) were male. There were 19 patients with dilated cardiomyopathy, 5 patients with dilated phase of hypertrophic cardiomyopathy, 13 patients with ischemic cardiomyopathy, 4 patients with cardiac sarcoidosis, 1 patient with cardiac amyloidosis, and 3 patients with hypertensive heart disease. The median eGFR was 35.8 (29.7, 47.7) mL/min/1.73 m^2^, and the median log-transformed plasma BNP level was 2.35 (2.07, 2.65) pg/mL. The median left ventricular end-diastolic diameter was 60 (55, 65) mm, and the LVEF was 38% (34%, 45%); all participants had LVEF < 50%. All patients attempted titration of GDMT. As a result, most patients received all four guideline-directed therapies. Notably, all patients received renin–angiotensin system inhibitors, consisting of 38 receiving angiotensin receptor neprilysin inhibitors and 5 receiving angiotensin-converting enzyme inhibitors due to hypotension.

### 3.2. Number of Heart Failure Hospitalizations

**Figure 1:**
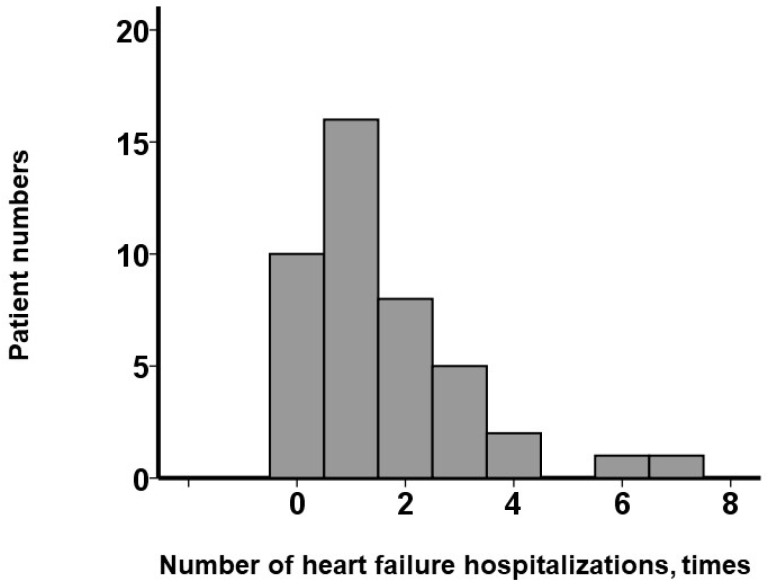
The distribution of heart failure hospitalization instances. The numbers of instances of previous heart failure hospitalization are widely distributed. Twenty-six (60%) patients had two or more heart failure hospitalizations before the initiation of vericiguat.

**Figure 2:**
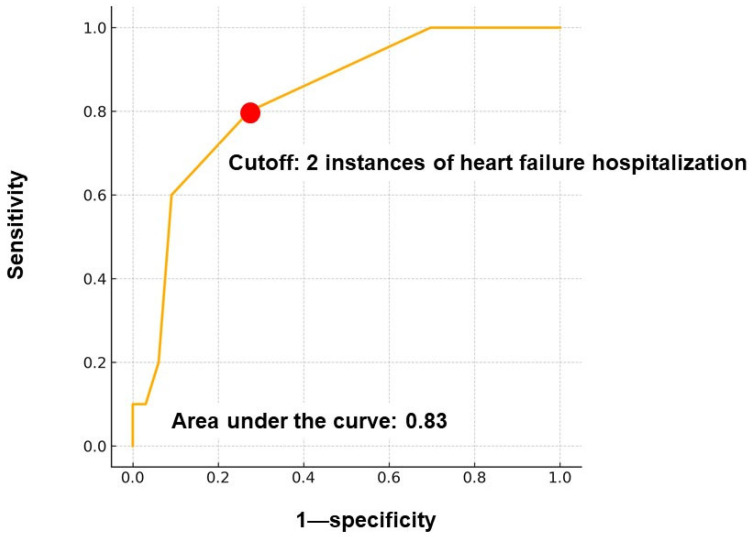
The time-dependent receiver operating characteristics analysis to determine the cutoff of heart failure hospitalization instances for predicting the primary outcomes. A total of 2 instances of heart failure hospitalization was identified as the cutoff.

As shown in Figure 1, 32 patients (74%) had experienced ≥ 1 instance of hospitalization, while 10 (23%) had experienced none. The maximum number of hospitalizations was 7, and the median was 1 (1, 3) time(s). Receiver operating characteristic analysis identified ≥ 2 prior heart failure hospitalizations as the optimal cutoff for predicting the primary outcomes (area under the curve = 0.83) (Figure 2).

### 3.3. Comparison Between Groups

Patients were stratified into two groups (≥2 vs. <2 prior hospitalizations). Their baseline characteristics are summarized in Table 1. The ≥2 hospitalizations group had a median of 3 (2, 4) hospitalizations, compared with 1 (0, 1) in the <2 group (*p* < 0.001). The ≥2 group tended to have more males, more severe anemia, higher BNP levels, and larger left atria, though not all differences reached statistical significance, except for left atrial size. Patients with <2 hospitalizations also received GDMT with a comparable prescription rate with their counterparts.

### 3.4. Prognostic Impact

During a median follow-up of 854 (415, 1118) days, 13 patients experienced the primary outcome—there were 6 deaths and 11 re-hospitalizations (4 patients experienced both death and re-hospitalization). Among the 11 re-hospitalization events, 5 were attributed to worsening of congestive heart failure, 2 were due to the development of new-onset atrial fibrillation, 2 were associated with progression of valvular heart disease, and 2 had unclear etiologies. During multivariable Cox regression analysis, ≥2 hospitalizations was an independent predictor (hazard ratio = 8.43; 95% confidence interval: 1.79–39.7; *p* = 0.007) after adjusting for hemoglobin and log-transformed BNP levels, both of which were also significant during the univariable analyses (Table 2).

**Figure 3:**
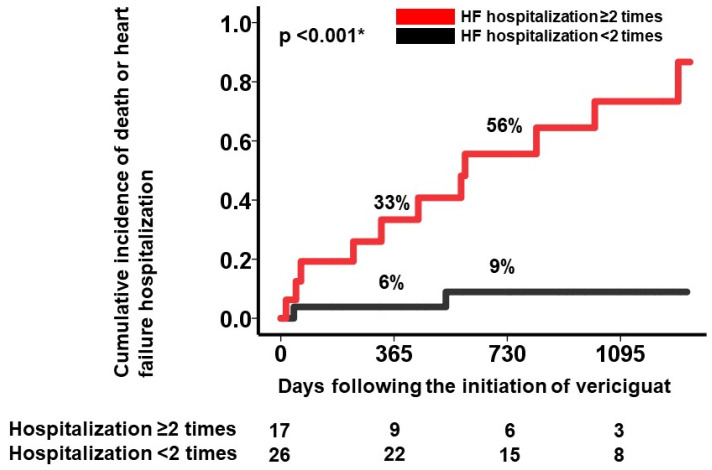
The cumulative incidence of the primary outcomes during vericiguat therapy, stratified by the number of heart failure hospitalizations. The cumulative incidence of the primary outcomes was significantly higher in patients with ≥2 previous instances of heart failure hospitalization compared with their counterparts. * *p* < 0.05 by the log-rank test.

The cumulative incidence of the primary outcome was significantly higher in patients with ≥2 hospitalizations compared with their counterparts (1-year: 33% vs. 6%; 2-year: 56% vs. 9%, *p* < 0.001; Figure 3).

When the patients were divided into two groups according to the median value of the logarithm of plasma BNP levels (2.3 pg/mL), a total of ≥2 hospitalizations was significantly associated with the primary outcome among those with BNP > 2.3 pg/mL (*p* = 0.023), but not among those with BNP ≤ 2.3 pg/mL (*p* = 0.63). When the patients were divided into two groups according to the median value of LVEF (38%), a total of ≥2 hospitalizations was significantly associated with the primary outcome among both those with LVEF < 38% (*p* = 0.046) and those with LVEF ≥ 38% (*p* = 0.025).

### 3.5. Trajectory of Clinical Parameters During 6-Month Follow-Up

During the six-month therapeutic period, the prevalence of heart failure medications remained unchanged in the whole cohort (*p* = 1.0 for beta-blockers, renin–angiotensin system inhibitors, and mineralocorticoid receptor antagonists; *p* = 0.50 for sodium–glucose co-transporter 2 inhibitors).

At six months following the initiation of vericiguat, the prescription rates of the following heart failure medications were not significantly different between the patients with ≥2 hospitalizations and those with <2 hospitalizations: renin–angiotensin system inhibitors (12/12 versus 23/23, *p* = 1.0), beta-blockers (12/12 versus 22/23, *p* = 0.46), mineralocorticoid receptor antagonist (12/12 versus 21/23, *p* = 0.29), and sodium–glucose co-transporter 2 inhibitor (11/12 versus 21/23, *p* = 0.97).

**Figure 4:**
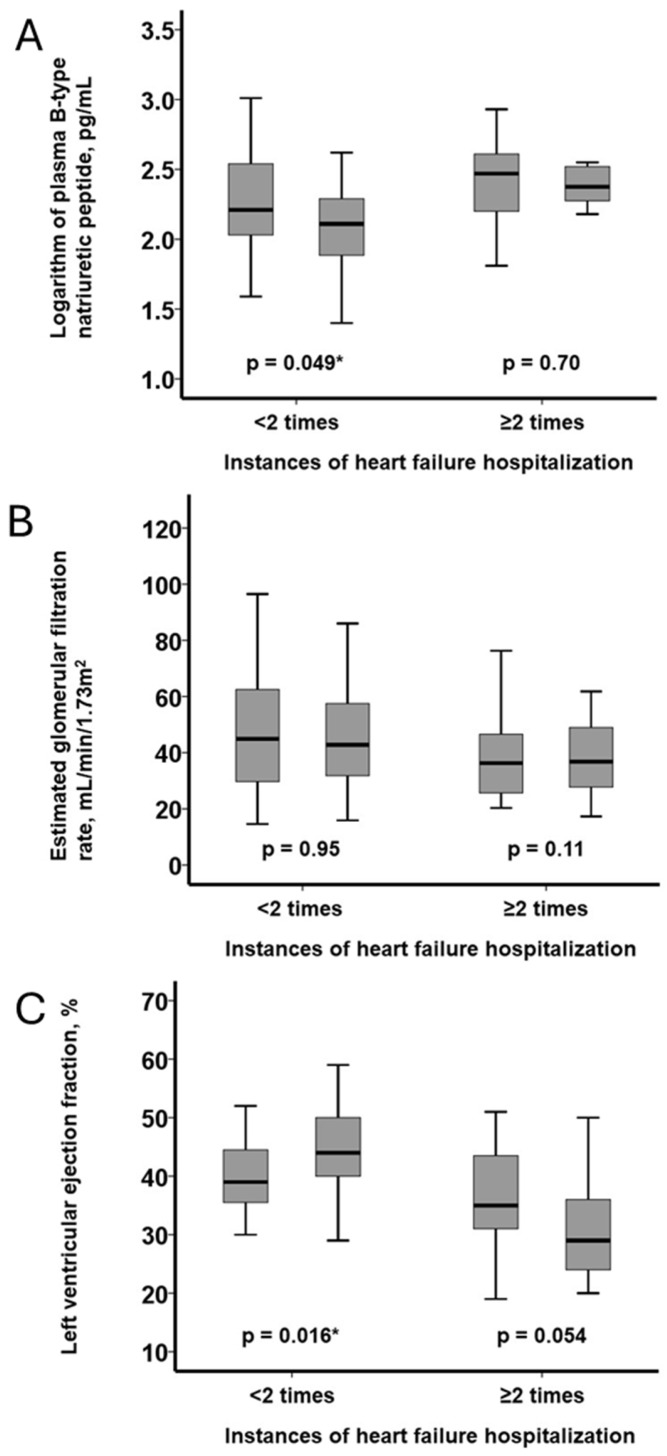
The trajectories of biomarkers and structural parameters at baseline and at six months following the initiation of vericiguat: the logarithm of plasma B-type natriuretic peptide (**A**), the estimated glomerular filtration rate (**B**), and the left ventricular ejection fraction (**C**). The trajectories of these parameters were stratified by the number of instances of heart failure hospitalization. * *p* < 0.05 by the Wilcoxon signed-rank test between the timings.

In patients with <2 hospitalizations, plasma BNP decreased significantly (*p* = 0.049), whereas no significant change was observed in those with ≥2 hospitalizations (*p* = 0.70; Figure 4A). eGFR remained statistically unchanged in both groups (*p* = 0.11 and *p* = 0.95, respectively; Figure 4B). LVEF improved significantly in the <2 group (*p* = 0.016) and tended to worsen in the ≥2 group (*p* = 0.054; Figure 4C).

**Figure 5:**
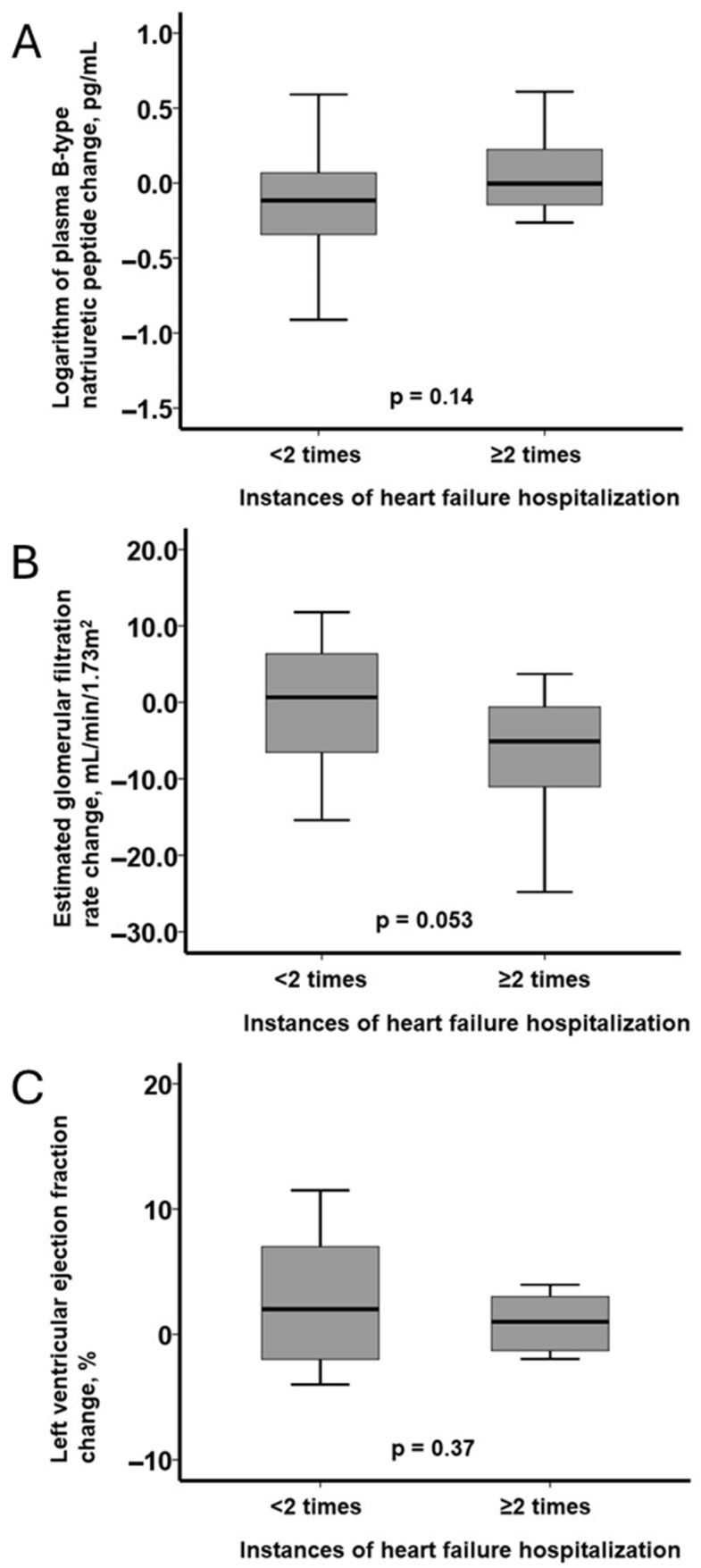
The changes in biomarkers and structural parameters at baseline and at six months following the initiation of vericiguat: the logarithm of plasma B-type natriuretic peptide (**A**), the estimated glomerular filtration rate (**B**), and the left ventricular ejection fraction (**C**). The changes in these parameters were compared between the two groups stratified by the number of instances of heart failure hospitalization. The comparison was performed by the Mann–Whitney U test between the groups.

Intergroup comparisons of parameter changes showed similar trends, though they did not reach statistical significance (BNP: *p* = 0.14; eGFR: *p* = 0.053; LVEF: *p* = 0.37; Figure 5A–C).

## 4. Discussion

In this retrospective analysis of real-world patients with heart failure with reduced/mildly reduced ejection fraction who underwent vericiguat therapy, we found that the number of prior heart failure hospitalizations was a powerful determinant of subsequent clinical outcomes during vericiguat therapy. The principal finding of our study is that a history of two or more hospitalizations is a significant and independent predictor of the primary composite endpoint of all-cause mortality or heart failure re-admission in patients receiving vericiguat therapy. After multivariable adjustment for key prognostic factors, including hemoglobin and plasma BNP levels, patients with two or more prior hospitalizations had an over eight-fold higher risk of adverse events compared to those with fewer than two hospitalizations. This finding suggests that, by the time a patient has been hospitalized twice for heart failure, they may have crossed a clinical threshold into a state of profoundly high risk, in which even the addition of a potent therapy like vericiguat may not substantially alter the disease trajectory. This concept is supported by evidence indicating that each HF hospitalization is not an isolated event, but rather an accelerator of disease progression, contributing to a vicious cycle of further myocardial injury, renal dysfunction, and neurohormonal activation [11]. Moreover, recurrent hospitalizations are strongly associated with increased mortality and substantial healthcare burden [12].

This prognostic divergence is strongly corroborated by the trajectory of key biological and structural markers. In the group with fewer than two prior hospitalizations, vericiguat therapy was associated with favorable cardiac reverse remodeling, evidenced by a significant reduction in plasma BNP levels and a significant improvement in LVEF over 6 months despite other heart failure medications remaining unchanged. These findings are consistent with recent real-world data from Japan, such as the study by Fuji et al., which also demonstrated that vericiguat significantly improves left ventricular reverse remodeling [13].

In contrast, patients with a history of two or more hospitalizations exhibited no such statistically significant beneficial response. Interestingly, although baseline plasma BNP level was a significant predictor in the univariable analysis, its prognostic value diminished after multivariable adjustment, in which the number of prior hospitalizations remained a robust independent predictor. This observation underscores the limitations of relying solely on natriuretic peptides for risk stratification in this setting. Given that plasma BNP levels are influenced by acute hemodynamic shifts, intravascular volume status, and renal function, they may not adequately capture the chronic, cumulative burden of disease that repeated hospitalizations reflect [14]. These findings highlight the added prognostic value of a simple clinical history metric over a single biomarker-based assessment.

Our findings do not contradict the results of the VICTORIA trial, but rather help to refine their clinical application. While the VICTORIA study established the efficacy of vericiguat in high-risk patients with worsening heart failure, our study identifies a simple, clinically intuitive metric—the number of prior hospitalizations—to stratify risk and potential therapeutic response even further. The notion that vericiguat is more effective in less advanced stages of disease is consistent with the VICTORIA study’s subgroup analysis, which showed a greater benefit in patients with lower NT-proBNP levels [9]. Our data, using hospitalization history as a surrogate for disease burden, support this observation.

The clinical implications of our study are substantial. The current guidelines recommend considering vericiguat for chronic heart failure patients who experience worsening heart failure despite optimal foundational therapy [1,10]. However, therapeutic inertia may result in delayed initiation, with vericiguat often reserved for patients with multiple prior hospitalizations [15]. Our results challenge this approach, suggesting that waiting until the second hospitalization may mean missing a critical therapeutic window. These findings support a paradigm shift toward earlier consideration of vericiguat, potentially after the first significant heart failure event. Furthermore, vericiguat’s neutral effect on renal function and serum potassium levels, as demonstrated in the VICTORIA trial and its sub-analyses, underscore its suitability for earlier introduction [16,17]. In clinical practice, the up-titration of foundational GDMT, particularly renin–angiotensin system inhibitors and MRAs, is often hindered by concerns about drug-related worsening renal function and hyperkalemia, particularly in high-risk patients [18,19]. In scenarios where the optimization of other GDMT is challenging, vericiguat, which can be relatively safely introduced, emerges as a particularly attractive option for early intervention to reduce residual risk. Early intervention could interrupt the cycle of decompensation and allow for more favorable biochemical and structural outcomes, as seen in our group with fewer than two hospitalizations, as well as other real-world studies [13,20].

## 5. Limitations

This study has several important limitations that warrant careful consideration. First, the retrospective and single-center nature of the study inherently limits its external validity. Institutional practice patterns and patient characteristics may not reflect broader populations, thereby constraining the generalizability of our findings. Second, the relatively small sample size and modest number of clinical events reduce statistical power, increase the risk of type II errors, and limit the reliability of multivariable modeling. As a result, residual confounding cannot be fully excluded despite statistical adjustments.

Third, patient selection for vericiguat initiation was not randomized and may have been influenced by unmeasured clinical judgment factors, such as frailty, compliance, or socioeconomic conditions. This introduces potential selection bias and indication bias, which are inherent in observational studies.

Fourth, although we used the number of prior hospitalizations as a surrogate marker of disease severity, this metric may not fully capture the complex pathophysiology or staging of heart failure. Notably, the determination of heart failure hospitalization is subjective and generally at the discretion of the attending physicians.

Fifth, the follow-up duration was relatively short and may have missed long-term outcomes, including late mortality or ventricular remodeling. Finally, echocardiographic and laboratory data were not centrally adjudicated and may have been subject to measurement variability. Notably, echocardiography was performed by well-trained sonographers and the outcomes were validated by board-certified cardiologists.

## 6. Conclusions

In conclusion, a history of two or more heart failure hospitalizations prior to the initiation of vericiguat is a powerful and independent predictor of adverse long-term outcomes during vericiguat therapy. These findings, supported by divergent responses in plasma BNP level and LVEF, suggest that to maximize the benefits of vericiguat, clinicians should consider initiating this therapy relatively earlier in the disease course, before patients cross a threshold of recurrent hospitalizations from which they may derive limited further benefit.

## Figures and Tables

**Table 1:** The baseline characteristics.

	Total (N = 43)	HF Hospitalization ≥2 Times (N = 17)	HF Hospitalization <2 Times (N = 26)	*p*-Value
Demographics				
Age, years	73 (60, 79)	74 (62, 80)	66 (56, 79)	0.52
Male	35 (81%)	12 (71%)	23 (88%)	0.14
Body mass index, kg/m^2^	22.9 (21.0, 25.1)	22.9 (21.2, 23.5)	23.0 (21.0, 26.4)	0.90
Number of heart failure hospitalizations, times	1 (1, 3)	3 (2, 4)	1 (0, 1)	<0.001 *
Comorbidity				
Diabetes mellitus	19 (44%)	8 (47%)	11 (42%)	0.50
Hypertension	23 (53%)	8 (47%)	15 (58%)	0.36
Dyslipidemia	22 (51%)	10 (59%)	12 (46%)	0.31
Atrial fibrillation	21 (49%)	10 (59%)	11 (42%)	0.23
Laboratory data				
Hemoglobin, g/dL	12.5 (11.2, 13.9)	12.5 (1.9, 13.4)	14.1 (11.9, 14.7)	0.051
Serum albumin, g/dL	3.7 (3.3, 4.1)	3.8 (3.3, 4.1)	3.7 (3.3, 4.1)	0.65
Serum sodium, mEq/L	139 (137, 141)	139 (137, 142)	140 (138, 140)	0.92
Serum potassium, mEq/L	4.5 (4.3, 4.8)	4.4 (4.3, 4.6)	4.5 (4.1, 4.9)	0.95
Estimated glomerular filtration rate, mL/min/1.73 m^2^	35.8 (29.7, 47.7)	36.3 (25.7, 46.6)	44.9 (29.7, 62.5)	0.40
Logarithm of plasma B-type natriuretic peptide, pg/mL	2.35 (2.07, 2.65)	2.47 (2.20, 2.61)	2.21 (2.03, 2.54)	0.16
Echocardiography				
Left atrial diameter, mm	46 (40, 52)	52 (47, 57)	41 (37, 46)	0.001 *
Left ventricular end-diastolic diameter, mm	60 (55, 65)	63 (56, 68)	58 (55, 64)	0.15
Left ventricular ejection fraction, %	38 (34, 45)	35 (27, 44)	39 (36, 45)	0.22
Medication				
Beta-blockers	41 (95%)	16 (94%)	25 (96%)	0.76
Renin–angiotensin system inhibitors	43 (100%)	17 (100%)	26 (100%)	1.0
Mineralocorticoid receptor antagonists	37 (86%)	14 (82%)	23 (88%)	0.57
Sodium–glucose co-transporter 2 inhibitors	35 (81%)	13 (76%)	22 (85%)	0.50

The baseline characteristics that were retrieved during vericiguat initiation are displayed. Data were compared between two groups that were divided by their history of heart failure hospitalization into a group that had been hospitalized ≥ 2 times and a group that had not. Continuous variables were recorded as medians (25% interquartile, 75% interquartile) and compared between the two groups by the Mann–Whitney U test. Categorical variables were recorded as numbers (percentages) and compared between the two groups by Fisher’s exact test. * *p* < 0.05.

**Table 2:** The variables associated with all-cause death or heart failure re-admission during vericiguat therapy.

	Univariable Analysis		Multivariable Analysis	
	Hazard Ratio (95% CI)	*p*-Value	Hazard Ratio (95% CI)	*p*-Value
Heart failure hospitalization ≥ 2 times	10.9 (2.41–49.4)	0.002 *	8.43 (1.79–39.7)	0.007 *
Age, years	1.06 (0.97–1.05)	0.80		
Atrial fibrillation	1.08 (0.36–3.24)	0.90		
Renin–angiotensin system inhibitors	0.17 (0.02–1.42)	0.17		
Beta-blockers	0.37 (0.05–2.89)	0.34		
Mineralocorticoid receptor antagonists	0.80 (0.20–3.19)	0.75		
Sodium–glucose co-transporter 2 inhibitors	0.29 (0.09–0.98)	0.047		
Hemoglobin, g/dL	0.49 (0.32–0.74)	0.001 *	0.51 (0.31–0.84)	0.008 *
Estimated glomerular filtration rate, mL/min/1.73 m^2^	0.97 (0.94–1.01)	0.081		
Logarithm of plasma B-type natriuretic peptide, pg/mL	10.4 (1.81–59.4)	0.009 *	3.39 (0.55–20.8)	0.19
Left ventricular ejection fraction, %	1.01 (0.94–1.08)	0.85		
Left atrial diameter, mm	1.10 (1.03–1.18)	0.038		

The baseline characteristics that were considered to be potentially associated with the clinical outcomes were included in the univariable analyses. Variables with *p* < 0.010 in the univariable analyses were included in the multivariable analysis with a forced method. CI, confidence interval. * *p* < 0.010.

## Data Availability

Data are available from the corresponding author upon reasonable request.
